# DNA Vaccine for West Nile Virus Infection in Fish Crows (*Corvus ossifragus*)

**DOI:** 10.3201/eid0909.030025

**Published:** 2003-09

**Authors:** Michael J. Turell, Michel Bunning, George V. Ludwig, Brian Ortman, Jeff Chang, Tully Speaker, Andrew Spielman, Robert McLean, Nicholas Komar, Robert Gates, Tracey McNamara, Terry Creekmore, Linda Farley, Carl J. Mitchell

**Affiliations:** * U.S. Army Medical Research Institute of Infectious Diseases, Fort Detrick, Maryland, USA; † U.S. Air Force, Fort Detrick, Maryland, USA; ‡ Centers for Disease Control and Prevention, Fort Collins, Colorado, USA; § Temple University, Philadelphia, Pennsylvania, USA; ¶ Harvard School of Public Health and the Center for International Development at Harvard University, Boston, Massachusetts, USA; # U.S. Department of Agriculture, Fort Collins, Colorado, USA; ** Wildlife Conservation Society, Bronx, New York, USA; †† Wyoming Department of Health, Laramie, Wyoming, USA; ‡‡ American Bird Conservancy, Washington, D.C., USA; 1 Drs. Turell and Bunning are co-lead authors of this article.

**Keywords:** vaccine, West Nile virus, fish crow, DNA vaccine, flavivirus

## Abstract

A DNA vaccine for West Nile virus (WNV) was evaluated to determine whether its use could protect fish crows (*Corvus ossifragus*) from fatal WNV infection. Captured adult crows were given 0.5 mg of the DNA vaccine either orally or by intramuscular (IM) inoculation; control crows were inoculated or orally exposed to a placebo. After 6 weeks, crows were challenged subcutaneously with 10^5^ plaque-forming units of WNV (New York 1999 strain). None of the placebo inoculated–placebo challenged birds died. While none of the 9 IM vaccine–inoculated birds died, 5 of 10 placebo-inoculated and 4 of 8 orally vaccinated birds died within 15 days after challenge. Peak viremia titers in birds with fatal WNV infection were substantially higher than those in birds that survived infection. Although oral administration of a single DNA vaccine dose failed to elicit an immune response or protect crows from WNV infection, IM administration of a single dose prevented death and was associated with reduced viremia.

West Nile virus (WNV), a mosquito-borne flavivirus, was recognized for the first time in the Western Hemisphere during summer 1999 in New York City and was associated with human, equine, and avian deaths (*1*–*4*). This virus is transmitted by a variety of mosquito species, mostly in the genus *Culex* (*5*–*7*). The New York 1999 strain of WNV differed genetically from other known strains of WNV except for an Israeli strain isolated from a dead goose in Israel in 1998 (*1*). With the exception of a laboratory study in Egypt involving hooded crows (*Corvus corone*) and house sparrows (*Passer domesticus*) (*8*), only these two nearly identical strains are known to kill birds (*9*,*10*). In 2000, WNV was detected in >4,000 bird carcasses in the United States (*11*), and the overall mortality rate was considered much greater. Several deaths attributed to WNV in the United States have occurred in valuable captive birds in zoologic collections (*12*). Currently, no treatment or vaccine is available for susceptible birds.

Vaccination may protect birds from lethal WNV infections. Accordingly, we examined a DNA vaccine developed for use in horses (*13*) for its ability to protect crows, a species known to be highly susceptible to lethal infection with this virus (*8*,*10*).

## Materials and Methods

### Vaccine

The plasmid DNA, pCBWN, codes for the prM and E glycoproteins of WNV. The plasmid was purified from *Escherichia coli* XL-1 blue cells with EndoFree Plasmid Giga Kits (QIAGEN, Inc., Santa Clarita, CA) and suspended in 10 mM Tris buffer, pH 8.5, at a concentration of 10.0 mg/mL. For IM vaccination, the DNA vaccine was formulated in phosphate-buffered saline (PBS), pH 7.5, at a concentration of 1.0 mg/mL. For oral exposure, the dry-microencapsulated DNA was suspended in PBS, pH 7.5, at a concentration of 2.0 mg/mL.

### Microencapsulation

The method for microencapsulating DNA was adapted from procedures previously described for virus and subunit vaccines and isolated proteins (*14*–*16*). We performed all steps with sterile reagents and aseptic technique. Two 10-mg aliquots of WNV cDNA were transferred to separate test tubes with enough water to make 9-mL volumes. Resulting suspensions were mixed on a clinical rotator until solution was complete; 1 mL of 0.6% w/v aqueous sodium alginate (Fluka Chemical Co., Ronkonkoma, NY) solution was added to each tube, and the contents of each were gently inverted 20 times. Each DNA/alginate solution was pumped at 1.2 mL/min through a 76-μm orifice in a 1-mm internal diameter glass tube against the side of which a 20-KHz laboratory sonicator probe was firmly pressed. The emerging train of droplets was directed into a modified T-tube, through which a recirculated 40 mL of 0.25% w/v neutral aqueous spermine hydrochloride (Sigma-Aldrich Corp., St. Louis, MO) solution was pumped at 10 mL/min. A placebo microcapsule formulation was prepared by using alginate reagent without DNA. Resulting microcapsule suspensions were allowed to equilibrate for 30 min, pelleted at 500 x *g* for 20 min, and washed three times by decanting, suspending, and repelleting. Wash liquids were reserved for measuring the DNA that escaped encapsulation. Placebo and vaccine formulations and washes were frozen at –20°C and lyophilized overnight, then suspended in 5 mL of PBS to produce a final concentration of 2 mg/mL of the encapsulated DNA.

### Crows

Fish crows (*C. ossifragus*) were captured with a rocket-propelled net at various locations in Maryland. Birds were transported to a biosafety level 3 laboratory at the U.S. Army Medical Research Institute of Infectious Diseases, allocated into four groups, and placed in stainless steel cages (3–4 birds/cage); blood was collected for evidence of antibodies against flaviviruses. Birds were provided a mixture of cat and dog food ad libitum and water. This diet was supplemented with hardboiled eggs as well as vitamin supplements.

### Plaque Assay

Serial 10-fold dilutions of the blood samples from each crow were made in standard diluent (10% heat-inactivated fetal bovine serum in medium 199 with Earle’s salts, NaHCO_3_, and antibiotics). These samples were tested for infectious virus by plaque assay on Vero cells in 6-well plates (Costar, Inc., Cambridge, MA) as previously described (*17*), except that the second overlay, containing neutral red stain, was added 2 or 3 days after the first overlay.

### Plaque-Reduction Neutralization Assay

Serum samples were assayed for WNV-specific antibodies by using the plaque-reduction neutralization test (PRNT), as previously described (*18*). Briefly, each serum sample was diluted 1:10 in standard diluent (as above) and mixed with an equal volume of BA1 (composed of Hanks’ M-199 salts, 1% bovine serum albumin, 350 mg/L of sodium bicarbonate, 100 U/mL of penicillin, 100 mg/L of streptomycin, and 1 mg/L of fungizone in 0.05 M Tris, pH 7.6) containing a suspension of WNV (NY99-4132 strain) at a concentration of approximately 200 plaque-forming units (PFU)/0.1 mL, such that the final serum dilution was 1:20 and the final concentration of WNV (the challenge dose) was approximately 100 PFU/0.1 mL. After 1-h ncubation at 37°C, we added the serum/virus mixtures onto Vero monolayers in 6-well plates, 0.1 mL per well in duplicate. We determined the mean percentage of neutralization for each specimen by comparing the number of plaques that developed (see Plaque Assay section) relative to the number of plaques in the challenge dose, as determined by back titration. Preliminary samples were screened for antibodies to WNV in the same manner, as well as for neutralizing antibodies to St. Louis encephalitis virus, a closely related flavivirus that may cross-react serologically with WNV (*19*) and may partially protect against WNV infection (*20*).

### Experimental Design

The crows were placed in four groups: 1) those inoculated IM with vaccine, 2) those that had oral vaccine, 3) positive controls (i.e., those that received placebo inoculation and viral challenge), and 4) room controls (i.e., those that received placebo inoculation and placebo challenge). After an acclimatization period of approximately 1 month, the 10 crows in group 1 (9 fish crows and 1 American crow [C. brachyrhynchos*]*) were inoculated IM with 0.5 mg of the DNA vaccine in a total volume of 0.5 mL (0.25 mL in each breast). The 9 crows in group 2 (8 fish crows and 1 American crow) were given 0.5 mg of the encapsulated DNA vaccine orally in 0.25 mL of PBS, and 20 fish crows (groups 3 and 4) were each inoculated and orally exposed as above except that a placebo was used in place of the vaccine. Blood was collected weekly from the jugular vein and the serum tested for neutralizing antibodies to WNV. Six weeks after vaccination, all birds in groups 1, 2, and 3 were inoculated subcutaneously with 0.1 mL of a suspension containing 10^5^ PFU (10^6^ PFU/mL) of the 397-99 strain of WNV, which had been isolated from the brain of an American crow that died in New York City during the fall of 1999 and passaged once in Vero cells before use in this study. The crows in group 4 were inoculated with 0.1 mL of diluent. Three or four crows in each group were bled (0.1 mL) from the jugular vein each day; each bird was bled every third day. Blood samples were added to 0.9 mL of diluent + 10 U of heparin/mL. Blood samples were frozen at –70°C until tested for infectious virus by plaque assay.

## Results

### Serologic Response

While neutralizing antibodies developed in 5 of the 9 fish crows that received the vaccine by the IM route at the 80% neutralization level for WNV by 14 days after vaccination, neutralizing antibodies to WNV did not develop in any of the remaining fish crows (8 orally exposed to vaccine and 20 placebo-exposed) in the same time period (Table 1). An antibody response at the 78% level developed in one of the remaining IM-vaccinated fish crows. Thus, a serologic response developed in six (67%) of the nine fish crows that received the vaccine by the IM route. However, by day 42 after vaccination, none of these crows retained a response at the 80% neutralization level.

**Table 1:** Effect of route of administration of a DNA West Nile virus vaccine on the protection of fish crows from challenge with virulent West Nile virus

Treatment^a,b^	No. tested	% seropositive^c^	% viremic	Peak viremia^d^	% survival
Room control	10	0	0	<1.7 (0.0)	100
IM	9	56	67	2.9^b^ (0.4)	100
Oral	8	0	88	5.2^c^ (0.8)	50
Placebo	10	0	100	4.3^c^ (0.3)	50

^a^IM, intramuscularly. Crows were inoculated IM with 0.5 mg of the DNA vaccine. Oral, crows were given 0.5 mg of the DNA vaccine orally. Placebo, crows were inoculated IM with 0.5 mg of nonspecific DNA and given 0.5 mg of nonspecific DNA orally.           ^b^Room controls were placebo inoculated and then challenged with diluent.           ^c^Percentage of crows whose serum produced >80% neutralization at 1:20 dilution.           ^d^Logarithm_10_ mean peak viremia in crows bled every third day after challenge (S.E.). No virus was detected in any of the room control birds and a value of 1.7 was assigned to birds from which no virus was detected for calculation of mean and S.E. Means followed by the same letter are not significantly different at α = 0.05 by student t test.

### Viremia Profiles and Survival

All of the mock-challenged crows survived. Similarly, all nine fish crows that received the IM vaccine survived (Table 1). However, 5 of 10 fish crows that received the placebo vaccine and 4 of 8 fish crows that received the oral vaccine died when challenged with virulent WNV. The difference in survival rates between the fish crows that received the IM vaccine and either of the other two groups was significant (Fisher exact test, p<0.03). A veterinary pathologist examined all crows that died during these studies, and signs typical of WNV infection in avian hosts (i.e., heart necrosis) were observed in all of these birds. (These data will be described in a separate article on WNV viral pathogenesis in fish crows.) Viremias were detected in all 10 crows that received the placebo inoculation, 7 of 8 fish crows that received the oral vaccine, and 6 of 9 fish crows that received the vaccine by the IM route (Table 1). Virus was not detected in any of the crows that received the placebo challenge. Mean logarithm_10_ peak viremia titers were significantly lower (T>2.75, df>15, p<0.017) in the fish crows that received the vaccine by the IM route (mean + S.E. = 2.9 + 0.4) than in fish crows that received the placebo vaccine (mean + S.E. = 4.3 + 0.3) or fish crows that received vaccine by the oral route (mean + S.E. = 5.2 + 0.8). The mean peak viremia titers for fish crows that received the placebo vaccine or the DNA vaccine by the oral route were not significantly different (T=1.1, df=16, p=0.287). In both the oral vaccine and placebo groups, fish crows that died had higher viremia than those that survived their infection with WNV (Table 2). Because birds were bled only every third day, accurately determining the duration of viremia in individual fish crows was not possible. Viremias were detected on days 1 to 6 after infection, and 9 of 10 birds that were viremic on day 1 were still viremic on day 4. However, only five of eight birds that were viremic on day 2 were still viremic on day 5, and only three of six birds that were viremic on day 3 were still viremic on day 6. No birds were viremic 7 days after infection. Thus, most viremias apparently lasted approximately 5 days during this study.

**Table 2:** Viremia levels in fish crows that survived or died after challenge with virulent West Nile virus

Treatment^a^	Survived (peak)		Died	
	No.	Viremia^b^	No.	Peak viremia^b^
IM	9	2.9 (0.4)	0	n/a
Oral	4	3.6 (0.7)	4	6.9 (1.0)
Placebo	5	3.8 (0.4)	5	4.8 (0.4)

^a^IM, intramuscularly. Crows were inoculated IM with 0.5 mg of the DNA vaccine. Oral, crows were given 0.5 mg of the DNA vaccine orally. Placebo, crows were inoculated IM with 0.5 mg of nonspecific DNA and given 0.5 mg of nonspecific DNA orally.           ^b^Logarithm mean peak viremia in crows bled every third day after challenge (S.E.). A value           of 1.7 was assigned to birds from which no virus was detected for calculation of mean and S.E.

## Discussion

Although the DNA vaccine failed to induce a long-lasting immune response, fish crows vaccinated with this vaccine by the IM route all survived challenge with virulent WNV. In contrast, oral administration of this vaccine failed to elicit an immune response, nor did it protect fish crows from a lethal challenge with WNV. The death rate in these crows (4 [50%] of 8), was identical to that observed in the placebo-vaccinated group (5 [50%] of 10) and in a second group of unvaccinated fish crows (4 [50%] of 8) tested later (M.J. Turell and M. Bunning, unpub. data). Although no deaths occurred in the IM-vaccinated fish crows, low-level viremia, consistent with that observed in the birds that survived their WNV infection in the other groups, did develop in six of the nine crows. Therefore, a single dose of the DNA vaccine did not elicit complete protection and sterile immunity to WNV infection. Additional studies need to be conducted with multiple doses of vaccination both by the IM as well as by the oral route to determine whether multiple doses might provide greater protection against WNV infection.

During the course of these studies, we determined that we had two American crows mixed in with the fish crows, one in the oral and one in the IM-vaccinated groups. High viremias (>10^6^ PFU/mL of blood) developed in both of these crows, and they died after challenge with virulent WNV. These data, based on a single bird in each group, were not included in the data presented in this report. Both hooded crows (*8*) and American crows (*10*) are highly susceptible to infection with WNV with nearly 100% case-fatality rates. In contrast, fish crows appear to be less likely to succumb to the infection.

The continued spread of WNV infection across the United States and reported deaths in raptors and rare captive birds in zoologic parks indicate the need to develop an effective avian vaccine for WNV. To break the transmission cycle, the vaccine must be able to substantially reduce the level of viremia below the level needed to infect a feeding mosquito, which is about 10^5^ PFU/mL of blood (*21*). By this standard, the vaccine performed reasonably well, with no vaccinated fish crow having a recorded viremia >10^4.7^. In contrast, 3 of 10 placebo-vaccinated fish crows had viremias >10^5^ PFU/mL of blood, and 5 of 10 had a peak viremia >10^4.8^ PFU/mL of blood. However, because the crows were bled only every third day, determining the actual peak viremias in these birds was not possible. If the goal of the vaccine is to protect rare and endangered avian species from death, rather than to prevent transmission, then the occurrence of a low-level viremia is not critical.
